# Herpes zoster and alopecia areata following mRNA BNT162b2 COVID‐19 vaccine: Controversial immune effects

**DOI:** 10.1111/jocd.15465

**Published:** 2022-10-31

**Authors:** Fabrizio Martora, Luigi Fornaro, Vincenzo Picone, Dario Marasca, Maurizio Gargiulo, Maria Carmela Annunziata, Gabriella Fabbrocini, Claudio Marasca

**Affiliations:** ^1^ Dermatology Unit, Department of Clinical Medicine and Surgery University of Naples Federico II Naples Italy; ^2^ Department of Dentistry University Hospital Federico II Naples Italy; ^3^ Unit of Maxillofacial Surgery, Department of Medicine and Surgery A. Cardarelli Hospital Naples Italy

**Keywords:** alopecia areata, COVID‐19, herpes zoster, vaccine

Dear Editor,

The Severe acute respiratory syndrome coronavirus 2 (SARS‐CoV‐2) pandemic spread over the world in 2019. Research advances have been produced vaccines to contain morbidity and mortality associated with COVID‐19 and in order to stop viral transmission.^1^ The Pfizer‐BioNTech‐162b2 is one of the most inoculated vaccines in Italy. Particularly, the mRNA‐162b2 vaccine is a lipid nanoparticle–encapsulated mRNA‐based vaccine that encodes the prefusion stabilized full‐length spike protein of the SARS‐CoV‐2.^1^ Due to vaccination, adverse effects were classified in early and delayed reactions. The first one usually occurs with messenger RNA (mRNA) COVID‐19 vaccines within the first 30 min after the vaccination and can likely be interpreted as immunoglobulin E‐mediated hypersensitivity. Delayed cutaneous adverse events reported are injection site inflammation (the most frequent one) followed by other rare reactions.^2^

To date in literature, there are reported few cases of delayed cutaneous reactions, such as delayed urticaria, an erythema multiforme like eruption, eczematous eruptions, generalized pruritic morbilliform, urticarial vasculitis and leukocytoclastic vasculitis.^2^ Both for immediate and delayed reactions it could be useful to perform skin testing to demonstrate the culprit role of vaccine.^2^ Few cases of bullous pemphigoid up to more unusual reactions, for example, erythromelalgia, pernio/chilblains, filling reactions and pityriasis rosea‐like rashes have also been reported.^3^, ^4^, ^5^

Here, we report a case of child who presented alopecia areata and herpes zoster.

after the second dose of the vaccine (Pfizer‐BioNTech‐162b2). At our clinical output, a 7‐year‐old girl presented with erythematous and vescicolous unilateral lesions localized mainly to the trunk associated with a burning sensation (Figure 1). Contextually, physical examination showed a nonscarring alopecic patch localized on occipital region with foci of hair regrowth. (Figure 2).

**FIGURE 1:**
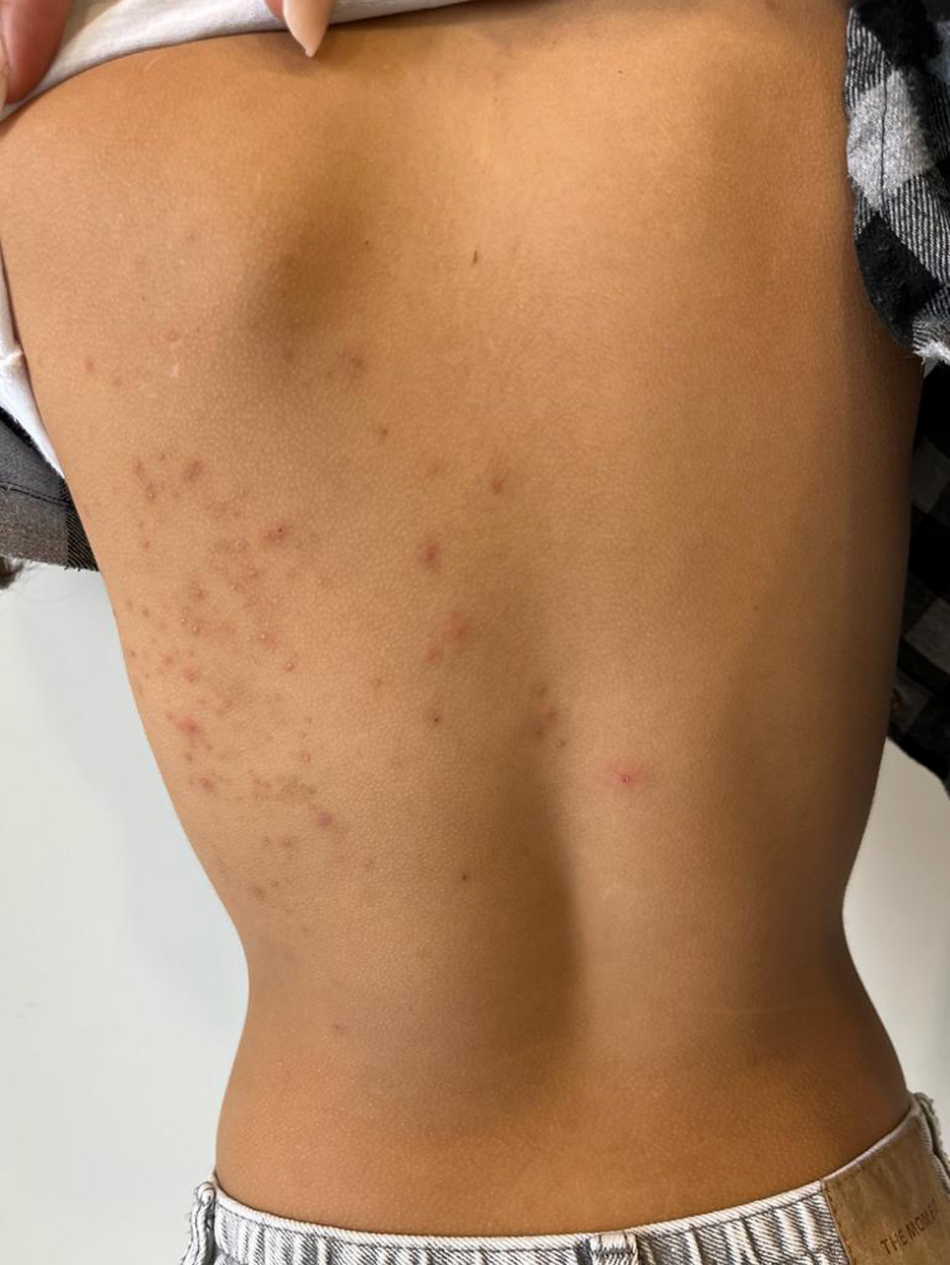
Erythematous and vescicolous unilateral lesions localized mainly to the trunk associated with a burning sensation

**FIGURE 2:**
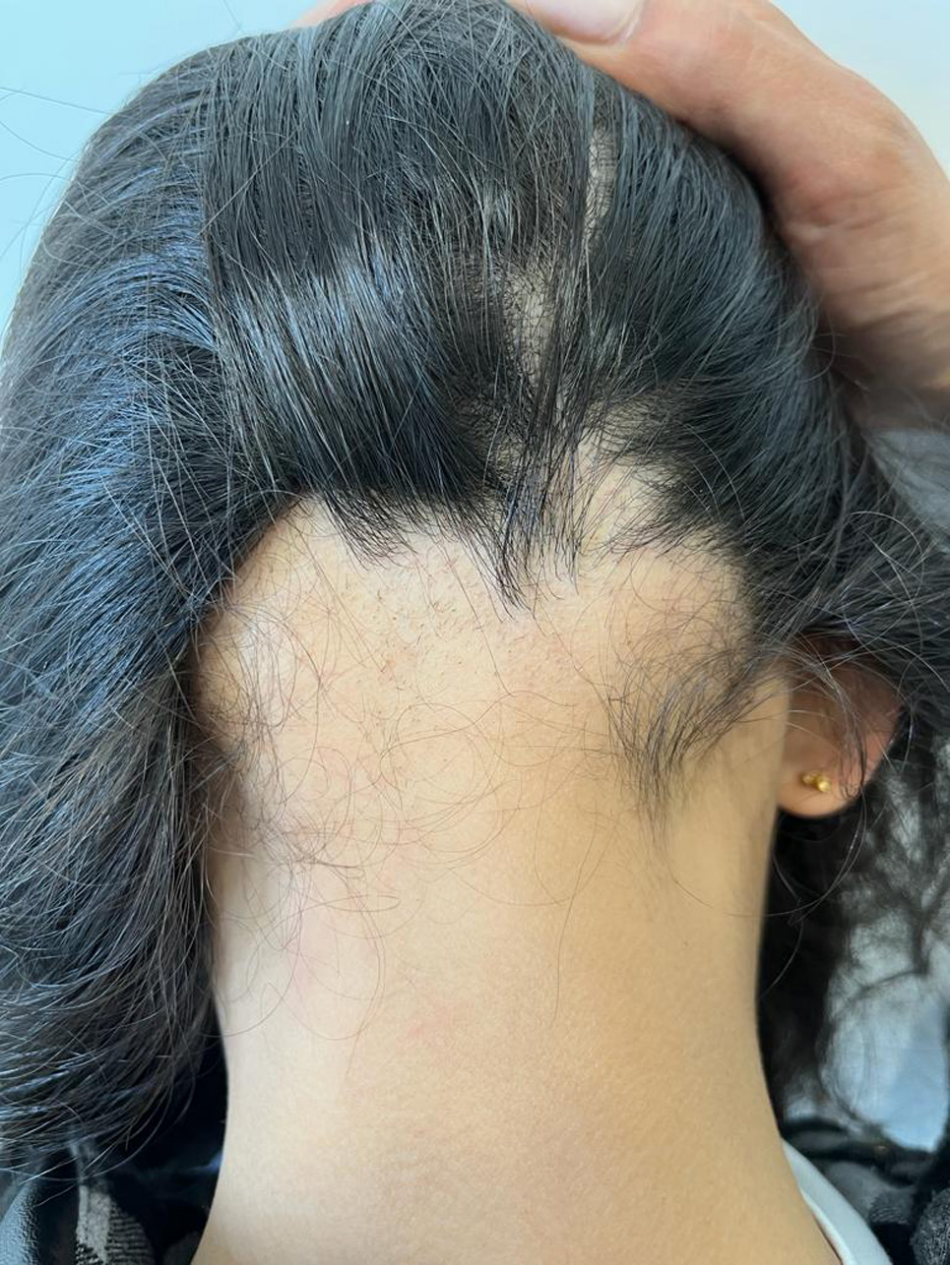
An alopecic patch patch localized on occipital region with foci of hair regrowth

During anamnesis, parents referred that both manifestations (alopecia areata and Herpes zoster) begin 20 days after the inoculation of the second dose of Pfizer‐162b2. Clinical history was negative for other disease, and she does not take drugs. Laboratory tests were performed (all negative), in particular we evaluated IgM for Varicella Virus which was negative.

The temporal correlation of the onset of these two diseases after vaccination and the absence of concomitant events and the laboratory tests negative allows us to relate these events. The underlying mechanisms that would explain the onset of these two diseases are still being studied: on the one hand probably, the COVID‐19 vaccine may cause some immunomodulation that allows VZV to escape from its latent phase,^6^ on the contrary, the occurrence of alopecia areata could be explained by a possible trigger role of vaccination leading to an autoimmune response in predisposed individuals as already described in the literature.^7^, ^8^

To our knowledge, there are no cases described in the literature reporting two reactions at the same time after vaccination. We would like to emphasize that we consider it essential and unavoidable that vaccination be carried out even though it may be related to some reactions such as our case because the efficacy and safety far outweigh the possible side effects. Certainly, further studies are still needed to confirm our hypothesis.

## AUTHOR CONTRIBUTIONS

**Martora Fabrizio**: data curation, formal analysis, investigation, visualization, writing‐original draft preparation, writing ‐ review & editing. **Luigi Fornaro**: conceptualization, validation, visualization, writing‐original draft preparation, writing ‐ review & editing. **Fabbrocini Gabriella**: conceptualization, validation, visualization, writing‐review & editing, supervision. **Vincenzo Picone**: data curation, formal analysis, investigation, visualization, writing‐original draft preparation, writing ‐ review & editing. **Dario Marasca:** data curation, formal analysis, investigation. **Maurizio Gargiulo:** data curation, formal analysis, investigation. **Maria Carmela Annunziata:** data curation, formal analysis, investigation. **Claudio Marasca:** data curation, formal analysis, investigation, visualization, writing‐original draft preparation, writing ‐ review & editing. All authors read and approved the final version of the manuscript.

## CONFLICT OF INTEREST

None to declare.

## ETHICAL APPROVAL

The authors confirm that the ethical policies of the journal, as noted on the journal's author guidelines page, have been adhered to. No ethical approval was required as this is a review article with no original research data.

## INFORMED CONSENT

The patients in this manuscript have given written informed consent to publication of their case details.

## Data Availability

Data sharing not applicable to this article as no datasets were generated or analyzed during the current study.

## References

[1] Francis AI , Ghany S , Gilkes T , Umakanthan S . Review of COVID‐19 vaccine subtypes, efficacy, and geographical distributions. Postgrad Med J. 2022;98(1159):389‐394. doi:10.1136/postgradmedj-2021-140654 37066438

[2] McMahon DE , Amerson E , Rosenbach M , et al. Cutaneous reactions reported after Moderna and Pfizer COVID‐19 vaccination: a registry‐based study of 414 cases. J Am Acad Dermatol. 2021;85(1):46‐55. doi:10.1016/j.jaad.2021.03.092 33838206 PMC8024548

[3] Martora F , Fabbrocini G , Marasca C . Pityriasis rosea after Moderna mRNA‐1273 vaccine: a case series. Dermatol Ther. 2022;35(2):e15225. doi:10.1111/dth.15225 34816549 PMC9286452

[4] Picone V , Martora F , Fabbrocini G , Marano L . "Covid arm": abnormal side effect after Moderna COVID‐19 vaccine. Dermatol Ther. 2022;35(1):e15197. doi:10.1111/dth.15197 34750923 PMC8646724

[5] Martora F , Picone V , Fornaro L , Fabbrocini G , Marasca C . Can COVID‐19 cause atypical forms of pityriasis rosea refractory to conventional therapies? J Med Virol. 2022;94(4):1292‐1293. doi:10.1002/jmv.27535 34931329

[6] Martora F , Fabbrocini G , Picone V . A case of herpes zoster ophthalmicus after third dose of Comirnaty (BNT162b2 mRNA) vaccine. Dermatol Ther. 2022;35(5):e15411. doi:10.1111/dth.15411 35220638 PMC9111833

[7] May Lee M , Bertolani M , Pierobon E , Lotti T , Feliciani C , Satolli F . Alopecia areata following COVID‐19 vaccination: vaccine‐induced autoimmunity? Int J Dermatol. 2022;61(5):634‐635. doi:10.1111/ijd.16113 35107173

[8] Martora F , Fabbrocini G , Nappa P , Megna M . Impact of the COVID‐19 pandemic on hospital admissions of patients with rare diseases: an experience of a southern Italy referral center. Int J Dermatol. 2022;61(7):e237‐e238. doi:10.1111/ijd.16236 35538737 PMC9347904

